# A Review on the Progressive Collapse of Reinforced Concrete Flat Slab–Column Structures

**DOI:** 10.3390/ma18092056

**Published:** 2025-04-30

**Authors:** Xiao Li, Tengfang Dong, Chengquan Wang, Weiwei Zhang, Rongyang Liu, Jingjing Wang

**Affiliations:** 1 Department of Civil Engineering, Hangzhou City University, Hangzhou 310015, China; lix@hzcu.edu.cn (X.L.); liuryzl@163.com (R.L.); 2 College of Civil Engineering and Architecture, Zhejiang University, Hangzhou 310058, China; 3 College of Civil Engineering, Fuzhou University, Fuzhou 350116, China; 240510024@fzu.edu.cn; 4 School of CML Engineering Architecture, Zhejiang Guangsha Vocational and Technical University of Construction, Dongyang 322100, China; wjjzugc@163.com

**Keywords:** flat slab–column structures, progressive collapse, experimental research, theoretical analysis, numerical simulation

## Abstract

Reinforced concrete flat slab–column structures, lacking the redundancy provided by a beam–column system, are susceptible to punching shear failure under extreme loading conditions, which may lead to progressive collapse with catastrophic consequences. A systematic review of recent advancements in the progressive collapse resistance of flat slab–column systems has been provided, categorizing the methodologies into experimental investigation, theoretical analysis, and numerical simulation. Experimental studies primarily utilize the Alternative Load Path methodology, incorporating both quasi-static and dynamic loading protocols to assess structural performance. Different column removal scenarios (e.g., corner, edge, and interior column failures) clarify the load redistribution patterns and the evolution of resistance mechanisms. Theoretical frameworks focus on tensile and compressive membrane actions, punching shear mechanism, and post-punching shear mechanism. Analytical models, incorporating strain-hardening effects and deformation compatibility constraints, show improved correlation with experimental results. Numerical simulations use multi-scale modeling strategies, integrating micro-level joint models with macro-level structural assemblies. Advanced finite element analysis techniques effectively replicate collapse behaviors under various column failure scenarios, validated by full-scale test data. This synthesis identifies key research priorities and technical challenges in collapse-resistant design, establishing theoretical foundations for future investigations of flat slab systems under multi-hazard coupling effects.

## 1. Introduction

Progressive collapse in structures refers to the phenomenon in which initial damage caused by accidental extreme loads propagates through structural components, triggering chain reactions that ultimately lead to either complete structural failure or a disproportionate large-scale collapse that exceeds the extent of the initial damage [1]. As a beamless floor system, reinforced concrete flat slab–column structures facilitate direct force transfer between columns and flat slabs, providing significant advantages in spatial efficiency and construction convenience. These characteristics have enabled their widespread use in commercial complexes, parking structures, and similar building types. However, their simplified load transfer pathways and low redundancy make the joint regions particularly vulnerable to punching shear failure. This inherent vulnerability significantly increases the potential risk of progressive collapse in such structural systems.

**Figure 1:**
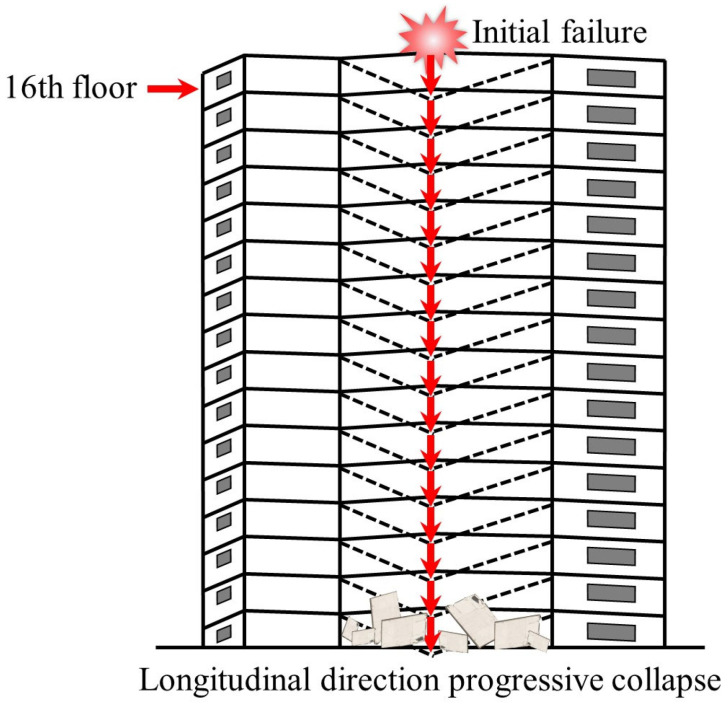
The progressive collapse of the 16th floor apartment, USA.

**Figure 2:**
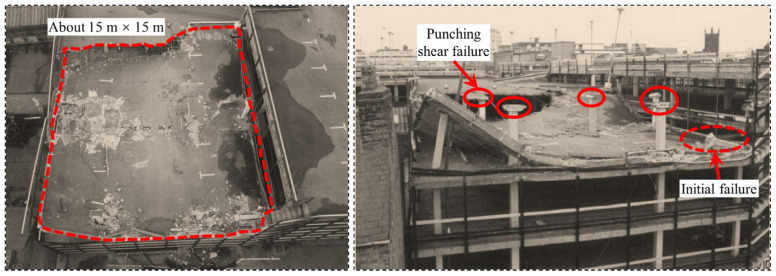
The site of the progressive collapse at the Pipers Row parking structure, UK [3].

Historically, two cases of progressive collapse in flat slab systems have been documented: (1) The 1971 collapse of a 16-story cast-in-place flat-plate apartment building on Federal Avenue, USA [2], as illustrated in Figure 1. The initial failure originated at the flat slab–column joint on the east side of the 16th floor, triggered by premature removal of roof shoring (resulting in mechanical room overload), compounded by substandard concrete (with compressive strength reaching only 60% of specified values), which exacerbated through-thickness cracking at the slab soffit. Forensic investigations identified three primary causes: inadequate rebar development length (30% below code requirements), improper concrete curing, and erroneous construction sequencing. (2) The 1997 collapse of the Pipers Row flat-plate parking structure in the UK [3], illustrated in Figure 2. The initiating event involved a punching shear failure at a corner column supporting a 15 m × 15 m roof slab. The original design, according to BS 8110 [4], underestimated edge joint shear loads by 20%, coupled with a concrete carbonation depth of 30 mm, which caused over 40% corrosion in top reinforcement and severe anchorage degradation. Post-collapse analysis revealed two critical factors: localized repairs that failed to address systemic deterioration, and the absence of periodic inspection protocols, which accelerated structural degradation. Additionally, notable cases include the 1973 collapse of the Skyline Plaza flat-plate apartment building in the United States [5] (due to shore failure during construction), the 1983 collapse of the Kimberley-Clark flat-plate warehouse in Canada [6] (due to insufficient consideration of extreme snow load scenarios in design), the 1987 collapse of the Morbio flat-plate shopping complex in Switzerland [7] (caused by a 35% reduction in joint shear capacity due to concrete spalling from freeze–thaw cycles), and the 2021 collapse of the Surfside flat-plate condominium in the United States [8] (due to a 50% cross-sectional loss in reinforcement of ground-floor columns caused by long-term seawater erosion). These incidents collectively highlight inherent limitations in conventional design codes regarding the prediction of extreme loads, material durability assessment, and joint redundancy design [9,10]. These limitations have prompted the structural engineering community to shift focus from component-level analysis to holistic structural resilience evaluation, advancing the theoretical framework for progressive collapse prevention.

Research indicates that the flat slab–column structures resist progressive collapse through three primary mechanisms: tension–compression membrane action, joint punching shear mechanism, and post-punching shear mechanism [11]. Fully mobilizing these resistance mechanisms constitutes a practical approach for robustness-oriented design [12], significantly improving collapse resistance. However, the interactions between these resistance components remain unclear, and a systematic design evaluation framework is still lacking. Consequently, this study provides a comprehensive review of experimental, theoretical, and numerical simulation advancements in progressive collapse resistance of flat-plate systems, aiming to establish references for collapse-resistant engineering design.

## 2. Experimental Study on Progressive Collapse of Flat Slab–Column Structure

Progressive collapse-resistant design requires a comprehensive understanding of the characteristics of accidental actions to implement targeted mitigation strategies. However, empirical evidence shows the impracticality of predicting specific abnormal load configurations and affected zones during the structure’s service life [13], leading to the widespread adoption of the Alternative Load Path (ALP) methodology. This approach operates independently of initial triggering events, focusing instead on the load redistribution capacity of the residual structural system. The methodology involves (1) hypothetical removal of designated load-bearing components, (2) quantitative assessment of the remaining structure’s load-carrying performance, and (3) systematic evaluation of structural redundancy and collapse progression probabilities.

Current international codes, including the GSA Guidelines (2016) [14], UFC 4-023-03 (2016) [15], and Eurocode EN 1993-1-8 (2006) [16], mandate the implementation of ALP for high-risk structures, with enhanced requirements for essential facilities requiring ≥2.0 structural robustness indices under Column failure scenarios.

### 2.1. Types of Experimental Methods

Within the framework of the ALP methodology, progressive collapse testing of flat slab–column systems can be classified into two distinct loading regimes: quasi-static testing and dynamic testing.

#### 2.1.1. Quasi-Static Testing

**Figure 3:**
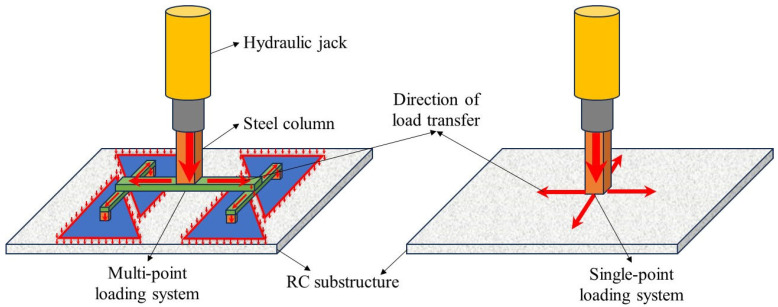
Loading device for quasi-static testing of flat slab–column structures.

Quasi-static testing protocols involve three sequential phases: (1) the pre-removal of designated column(s) to simulate initial local failure, (2) the anchorage of failed column stubs using reaction frames to simulate the actual boundary conditions of residual structures, and (3) the application of load-controlled or displacement-controlled loading protocols. As shown in Figure 3, two loading configurations exist: single-point loading (concentrated force application) and multi-point loading (simulating uniformly distributed loads). The single-point configuration directly applies concentrated loads at the removed column location, providing simpler implementation and operational convenience. Conversely, the multi-point configuration utilizes spreader beams and distribution plates to distribute concentrated loads across multiple application points. Qian et al. [17] conducted a comparative analysis of the progressive collapse resistance of flat-plate systems under two loading configurations. The experimental results revealed the secondary punching shear failure at the removed column location under single-point loading, while multi-point loading eliminated such localized failure modes, better simulating actual collapse mechanisms. This observation aligns with the US Department of Defense Unified Facilities Criteria [15], which recommends distributed load simulation for collapse testing. This methodology was further validated by Sasani et al. [18] and Sasani and Sagiroglu [19,20] through existing building tests, demonstrating rapid axial force dissipation above removed columns, while gravity-induced distributed loads dominate the structural downward movement. However, during the large deformation phases, non-uniform load distribution occurs due to geometric nonlinearity in slab deformation.

#### 2.1.2. Dynamic Testing

**Figure 4:**
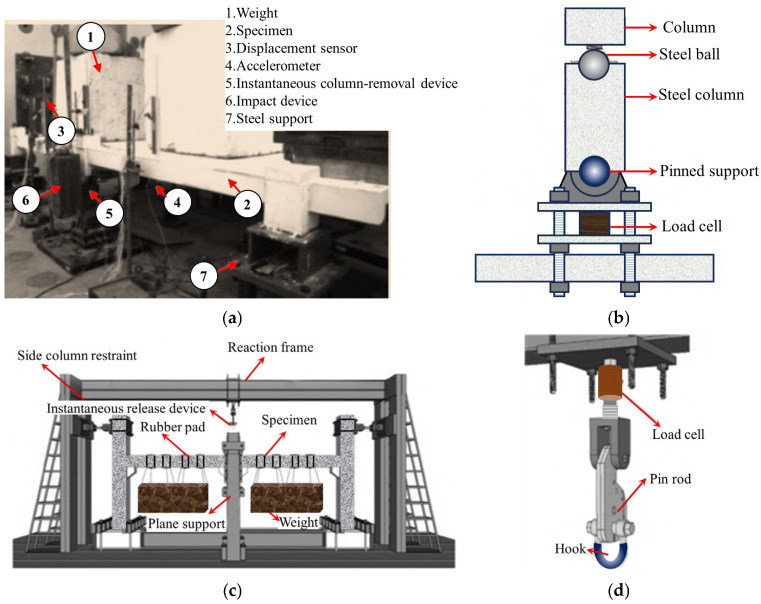
Loading device for dynamic testing of flat slab–column structures. (**a**) Install an ICRD from below [24]. (**b**) ICRD [24]. (**c**) Install a hook-based column-removal device from above [25]. (**d**) Hook-based column-removal device [25].

While the quasi-static testing methodology effectively captures the evolution of resistance mechanisms during collapse progression, forensic analyses of actual collapse incidents [21,22,23] reveal that initial structural damage predominantly occurs within milliseconds of abnormal dynamic actions. Static testing inherently fails to replicate the critical dynamic responses during the instantaneous column loss. Consequently, dynamic testing holds significant engineering importance. In a dynamic test, the distributed load under normal service conditions is first simulated by stacking or suspending heavy objects. Then, by removing a column instantaneously, the structural dynamic response and the dynamic process of load redistribution can be analyzed. To achieve instantaneous column removal, two equivalent methods are commonly adopted: (1) placing a vertical support beneath the failed column [24], with an instantaneous column-removal device (ICRD) that causes the support to lose its load-bearing capacity instantaneously, as shown in Figure 4a,b; and (2) installing a hook connection above the failed column [25], with the sudden release of the hook simulating instantaneous column failure, as illustrated in Figure 4c,d.

Qian et al. [26,27] quantitatively analyzed the dynamic responses of flat-plate systems under three failure scenarios: interior column loss (Case 1), simultaneous loss of interior and adjacent edge columns (Case 2), and corner column loss (Case 3). The results demonstrated that instantaneous column removal induces structural vibration, amplifying load increments on surviving columns to 124.8% of the original removed column load in Case 1 and 117.0% in Case 2. Furthermore, Qian’s experimental studies [26,27,28] on substructure dynamic testing revealed that multi-column failure scenarios produce 207% greater peak displacements compared to single-column removal. Peng et al. [29,30] conducted dynamic tests on single-story substructures, identifying that strain rate effects enhance rebar yield strength by 10% and concrete compressive strength by 15%, with a dynamic amplification factor (DAF) of 1.15 for total axial forces in adjacent columns. Adam et al. [31] performed full-scale dynamic testing on a two-story reinforced concrete flat-plate structure under corner column removal, recording a vertical displacement DAF of 2.6 and an axial load DAF of 1.24. Russell et al. [32] conducted seven 1:3-scaled reinforced concrete flat slab–column dynamic tests, observing successful load redistribution without flexural collapse in 85% of cases. However, punching shear failures triggered amplified dynamic responses, yielding a displacement DAF of 1.5. Kokot et al. [33] conducted nonlinear time-history analyses on full-scale systems, revealing structural survival with multiple plastic hinges and a DAF ranging from 1.72 to 1.87. Cuong et al. [34] tested edge and penultimate column-removal scenarios, reporting a DAF between 1.09 and 1.28 with negligible influence on ultimate failure modes.

### 2.2. Types of Column Failure Scenarios

**Figure 5:**
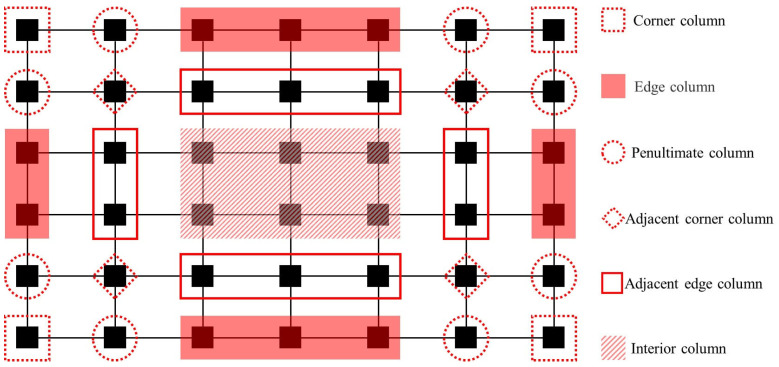
Standardized nomenclature for column failure locations.

In practical engineering scenarios, perimeter columns exhibit significantly higher vulnerability to impact-induced failure mechanisms caused by aircraft collisions, vehicular impacts, or rockfall events. Furthermore, abnormal extreme loads, including seismic actions, fire exposure, and blast loading, can induce simultaneous multi-column failure patterns through cascading failure mechanisms. Therefore, different accidental extreme loads can lead to various column failure scenarios, which, in turn, affect the structural resistance to progressive collapse. Figure 5 codifies the locations for progressive collapse analysis using standardized nomenclature.

#### 2.2.1. Load Redistribution Capability

The location of column failure exerts a significant influence on load redistribution mechanisms in flat-plate structures: (1) Interior column removal demonstrates superior load redistribution capacity. Xue et al. [35] revealed through pushdown analysis that 98% of the original column load is redistributed to four adjacent edge columns. Huang et al. [36] further quantified, via quasi-static testing, that edge columns initially bear 82% of redistributed loads, while 18% is borne by corner columns. At a 2.5% drift ratio, this distribution shifts to 51% (edge) and 49% (corner), indicating activation of progressive membrane action. (2) Edge column failure exhibits directional load transfer constraints. Yang et al. [37] identified predominant short-span load transfer to adjacent columns, with 60–65% of forces redirected along primary bending directions. Cuong et al. [34] quantified that 40% of the failed column load transfers to adjacent edge columns, while only 20% reaches corner columns, leaving 40% redistributed through the two-way slab action (which is unaccounted for in conventional design methods). (3) Corner column failure scenarios exhibit a 55–62% reduction in redistribution capacity due to boundary condition degradation, with 78% of cases demonstrating brittle rebar fracture at flat slab–column intersections [38].

**Figure 6:**
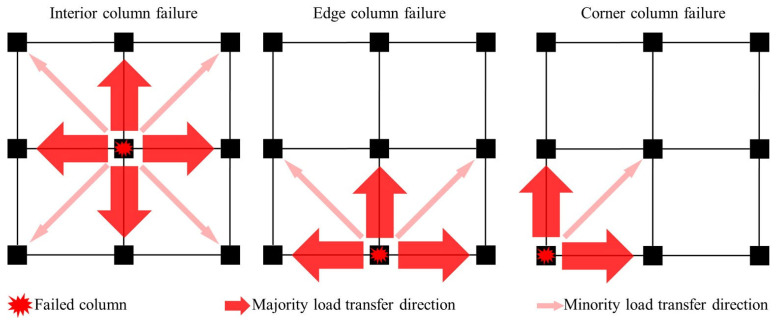
Load transfer mechanisms for different column failure scenarios.

A comparative analysis of failure modes reveals distinct load transfer mechanisms. Ma et al. [39] demonstrated similar force redistribution patterns between the edge column failure and combined edge-interior column failure scenarios, where adjacent edge columns and corner columns collectively sustain 83–91% of redistributed loads, while distant corner columns contribute less than 5%. Notably, load transfer mechanisms evolve dynamically through collapse progression. Qian et al. [17] quantified, through interior column removal tests, that compressive membrane action (CMA) dominates the initial stages, redistributing 74% of loads bidirectionally to adjacent columns. Post-punching failure, tensile membrane action (TMA) activates through rebar-anchorage mechanisms, with load distribution shifting to 19% (adjacent edge columns) and 6.4% (corner columns). These findings confirm that interior column failure scenarios benefit from superior redundancy, exhibiting higher collapse resistance than the perimeter column failures. Typical load redistribution pathways are schematically illustrated in Figure 6, highlighting critical differences in force transmission topology between failure modes.

#### 2.2.2. Resistance Curve and Failure Mode

**Figure 7:**
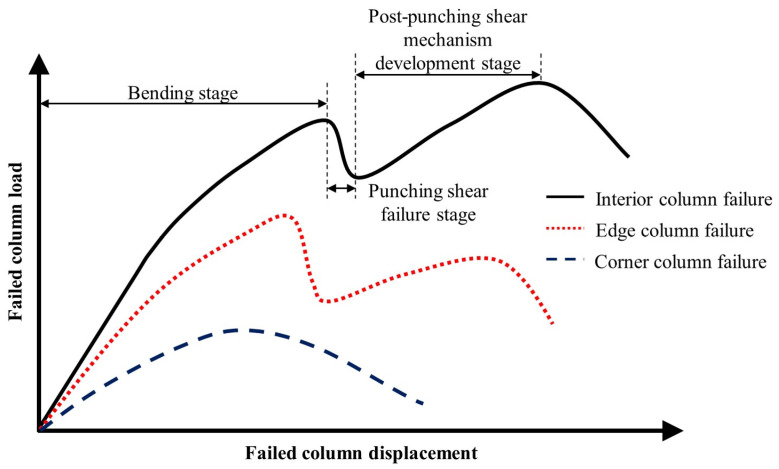
Load–displacement curves for different column failure scenarios.

**Figure 8:**
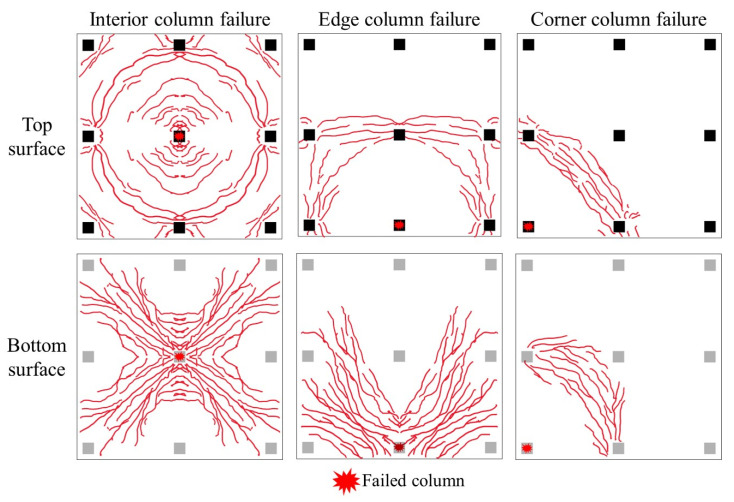
Failure modes for different column failure scenarios.

Based on experimental studies by various scholars [17,34,35,36,37,38,39], the load–displacement curve (Figure 7) and failure mode diagram (Figure 8) for the same flat slab–column structure under different column failure scenarios are summarized. The load–displacement curve can generally be divided into three stages: the bending stage, the punching shear failure stage, and the post-punching shear mechanism development stage. In the bending stage, the structure mainly relies on the bending mechanism of the slab and the CMA to resist collapse. When the punching shear stress near the joint reaches the concrete’s ultimate limit, the structure experiences punching shear failure, resulting in a sudden drop in resistance. If the structure includes complete through-column reinforcement or shear-resistant reinforcement, post-punching shear failure allows the development of the TMA to resist collapse, thereby increasing the resistance again. In the case of interior column failure, the surrounding frame can still provide strong boundary constraints, leading to a higher first peak load. After the punching shear failure, the structure can still develop the TMA, leading to a higher ultimate load. In contrast, when an edge column fails, the boundary constraints are relatively reduced, causing a decrease in both the first peak load and the ultimate load. Moreover, the structure’s ductility is weaker than in the interior column failure scenario. When a corner column fails, due to the lack of necessary boundary constraints, the structure cannot develop effective CMA and TMA. Consequently, the structure fails after reaching the first peak load, and the resistance consists of only one stage. In terms of failure modes, when an interior column fails, a ring-shaped crack forms on the upper surface of the slab. When an edge column fails, the crack forms a semi-ring around the failed edge column. When a corner column fails, most of the cracks are concentrated in the slab area around the corner column.

### 2.3. Types of Resistance Mechanisms

During the progressive collapse of flat slab–column structures, four primary resistance mechanisms are involved in resisting failure: the CMA, the TMA, the punching shear mechanism, and the post-punching shear mechanism [40]. Several experimental studies have revealed the operating principles of the resistance mechanisms.

Zhang [41] confirmed through quasi-static tests that the CMA plays a controlling role in final failure and suggested enhancing structural performance by optimizing boundary conditions and increasing continuous reinforcement. Keyvani et al. [42] found that when the interior column fails, the flat slab–column structure can generate a compression membrane force on the order of 700 kN/m. Park [43] and Regan [44] pointed out that the TMA can increase the ultimate resistance of the structure, but it must meet the ductility requirements, and rigid edge constraints are necessary for its development [45]. Qian [17] and Xue [35], through pushdown tests, demonstrated that the CMA dominates the resistance in the small deformation stage, while after punching shear failure, the TMA and pinning mechanism can still maintain 70% of the peak load as residual bearing capacity. Static load tests by Yi [46,47] and Huang [36] both validated the synergistic effect of the bending mechanism and membrane mechanism, emphasizing the importance of continuous reinforcement in enhancing the effectiveness of the TMA. Yang et al. [37,48] further found that when a corner column fails, there is a dynamic transition between the bending, tension membrane, and post-punching mechanisms.

It is crucial to emphasize that bending reinforcement plays a vital role in influencing the post-punching residual bearing capacity. Studies by Hawkins and Mitchell [9], Melo and Regan [49], and others, have demonstrated that when solely bending reinforcement is employed, the residual bearing capacity may reach as high as 30% of the peak value. However, when combined with tensile reinforcement and integrity reinforcement, the residual bearing capacity can increase to as high as 70% [7,11,49,50,51,52]. This provides a significant theoretical foundation for the design of flat slab–column structures capable of resisting progressive collapse.

## 3. Theoretical Analysis of Progressive Collapse in Flat Slab–Column Structures

### 3.1. Membrane Action

**Figure 9:**
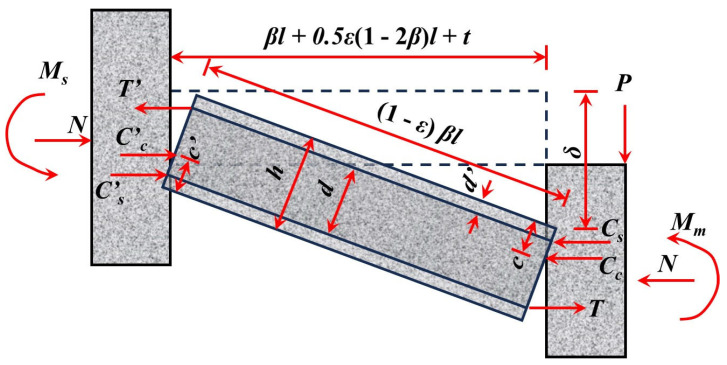
Deformation coordination diagram of the Park–Gamble compression membrane model [53].

**Figure 10:**
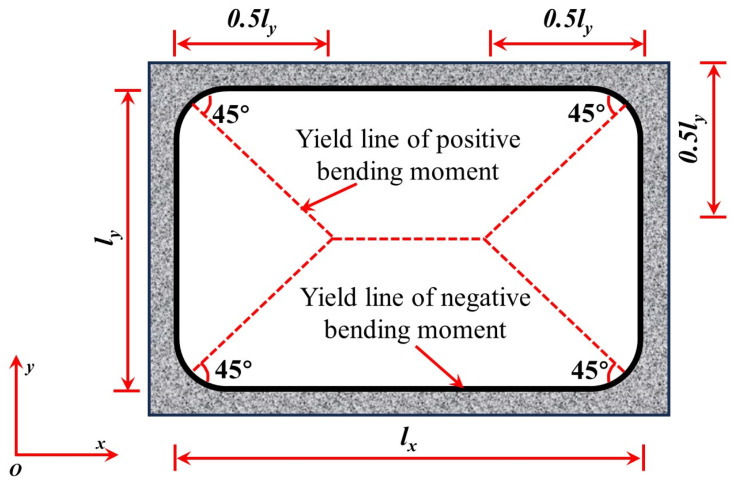
Yield line of the biaxial slab compression membrane model.

Park et al. [53] and Keenan [54] developed the Park–Gamble membrane action theoretical model based on the Rigid-Plastic Body assumption (Figure 9). This model aims to quantify the membrane action in slab–column connections, where *M_m_* represents the moment at the slab end near the interior column, while *M_s_* represents the moment at the slab end near the edge column. $C_{c}^{\prime }$ and *C_c_* denote the concrete pressures at the slab ends near the edge and interior columns, respectively. They also proposed Equation (1) to calculate the ultimate bearing capacity of the tensile membrane action, assuming full-section concrete cracking and the yielding of reinforcing bars [43]. However, one limitation of this model is its assumption of full yielding of reinforcing bars, which does not fully reflect the actual behavior in cases where the compressed reinforcing bars have not yet yielded. In subsequent studies, Park [55] expanded on this model by developing biaxial slab compression membrane models with fully constrained and partially constrained boundaries, as well as fully constrained tension membrane models (illustrated in Figure 10). These advancements address the effects of boundary constraints on membrane action and offer a more comprehensive understanding of how slab–column connections behave under load. While the models introduced by Park et al. provide valuable insight into the membrane action, further exploration of the effects of non-yielding reinforcement and different boundary conditions remains necessary for a more realistic prediction of collapse behavior.

(1) $$\frac{wl_{x}^{2}}{T_{x}\delta }=\frac{\pi^{3}}{4\sum_{n=1,3,\ldots }^{\infty }\left(\frac{1}{n^{3}}\right){\left(-1\right)}^{0.5(n-1)}\langle 1-{\left[\cosh\left(\frac{n\pi l_{y}}{2l_{x}}\sqrt{\frac{T_{x}}{T_{y}}}\right)\right]}^{-1}\rangle }$$

where *w* is the uniformly distributed load per unit area on the slab, *δ* is the central deflection corresponding to *w*, *l_x_* and *l_y_* are the clear spans in the two orthogonal directions, and *T_x_* and *T_y_* are the yield stresses of the reinforcing steel per unit width in the two orthogonal directions.

**Figure 11:**
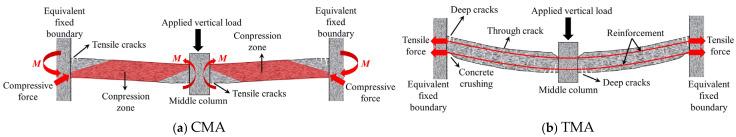
Schematic diagram of the tension and compression membrane formation mechanisms [58].

Following the Park–Gamble model, Hawkins and Mitchell [9] proposed the membrane analogy method (Equations (2)–(4)) for in-plane restrained slabs, assuming that failure occurs when the slab surface adopts a circular shape and the concrete has fully cracked. While this model provided early insights into membrane action, it oversimplifies the failure process by assuming complete cracking, not capturing more complex failure modes. Pham et al. [56] introduced a simplified version, suggesting that the slab’s ultimate strength is solely contributed by the top reinforcement in the hogging moment region. This approach, though useful, overlooks the effects of bottom reinforcement and boundary conditions. Bailey [57] developed a membrane model with a low reinforcement ratio for unrestrained slabs, but its application to typical slabs with higher reinforcement is limited. Building upon this, Qian et al. [58] optimized this model by incorporating experimental data (Figure 11), resulting in the punching shear strength equation (Equation (5)) and membrane analogy equations (Equations (7)–(12)). However, this model still assumes idealized reinforcement behavior and boundary conditions. These models highlight the progression of membrane analogy approaches, but further refinement is needed to address nonlinear concrete behavior and varying boundary effects.

(2) $$w=\frac{2T_{x}\sin\sqrt{6\varepsilon_{x}}}{l_{x}}+\frac{2T_{y}\sin\sqrt{6\varepsilon_{y}}}{l_{y}}$$

(3) $$\varepsilon_{y}=\frac{\varepsilon_{x}l_{x}^{2}}{l_{y}^{2}}$$

(4) $$\delta =\frac{3l_{x}\varepsilon_{x}}{2\sin\sqrt{6\varepsilon_{x}}}$$

where *w* is the uniformly distributed load per unit area to be predicted; *δ* is the mid-span deflection corresponding to *w*; *l_x_* and *l_y_* are the lengths in the short-span and long-span directions, respectively; *T_x_* and *T_y_* are the forces per unit length of reinforcing steel in the *x* and *y* directions, respectively; and *ε_x_* and *ε_y_* are the strains in the *x* and *y* directions, respectively.

(5) $$\begin{array}{c} P=\frac{1}{\beta l_{n}}\left\{0.85f_{c}^{\prime }\beta_{1}bh\left[\frac{h}{2}\left(1-\frac{\beta_{1}}{2}\right)+\frac{\delta }{4}\left(\beta_{1}-3\right)\right.\right.+\frac{\beta {\left(2l_{n}\right)}^{2}}{4\delta }\left(\beta_{1}-1\right)\left(\varepsilon +\frac{t}{l_{n}}\right)\\ +\frac{\delta^{2}}{8h}\left(2-\frac{\beta_{1}}{h}\right)\left.+\frac{\beta {\left(2l_{n}\right)}^{2}}{4h}\left(1-\frac{\beta_{1}}{2}\right)\left(\varepsilon +\frac{t}{l_{n}}\right)-\frac{\beta_{1}\beta^{2}{\left(2l_{n}\right)}^{4}}{16h\delta^{2}}{\left(\varepsilon +\frac{t}{l_{n}}\right)}^{2}\right]\\ -\frac{{\left(T^{\prime }-T-C_{s}^{\prime }+C_{s}\right)}^{2}}{3.4f_{c}^{\prime }b}+\left(C_{s}^{\prime }+C_{s}\right)\left(\frac{h}{2}-d^{\prime }-\frac{\delta }{2}\right)\left.+\left(T^{\prime }+T\right)\left(d-\frac{h}{2}+\frac{\delta }{2}\right)\right\} \end{array}$$

where *P* is the structural bearing capacity induced by the membrane compression mechanism; *b* is the width of the beam; *l_n_* is the span length of the beam; *β*_1_ is the ratio of the distance between the mid-span plastic hinge and the nearest support to 2*lₙ*; *δ* is the vertical displacement of the inner column; *h* is the depth of the beam; *T*′ and *T* are the tensile forces in the steel at the support and beam–column interface, respectively; *C*′ and *C* are the compressive forces in the concrete at the support and beam–column interface, respectively; *ε* is the strain caused by horizontal shrinkage and creep; *t* is the horizontal movement at the support; and the calculation formula for *ε* is shown in Equation (6).

(6) $$\varepsilon +\frac{t}{l_{n}}=\frac{\left(\frac{1}{hbE_{c}}+\frac{1}{l_{n}S}\right)\left[0.85f_{c}^{\prime }\beta_{1}b\left(\frac{h}{2}-\frac{\delta }{4}-\frac{T^{\prime }+T-C_{s}^{\prime }+C_{s}}{1.7f_{c}^{\prime }\beta_{1}b}\right)+C_{s}-T\right]}{1+\frac{0.85f_{c}^{\prime }\beta_{1}\beta l_{n}^{2}b}{\delta }\left(\frac{1}{hbE_{c}}+\frac{1}{l_{n}S}\right)}$$

where *E_c_* is the elastic modulus of concrete, *S* is the horizontal stiffness of the vertical support, and *S* = 93 kN/mm.

(7) $$T_{2}=\frac{bKT_{0}^{\mathrm{Bot}}}{2(1+k)}\frac{\sqrt{L^{2}+l^{2}}}{2}$$

(8) $$C=\frac{kbKT_{0}^{\mathrm{Bot}}}{2}\left(\frac{k}{1+k}\right)\frac{\sqrt{L^{2}+l^{2}}}{2}$$

(9) $$S=\frac{\left(C-T_{2}\right)}{\tan\varphi }=\frac{1}{\tan\varphi }\times \frac{bKT_{0}^{m}}{2}(k-1)$$

(10) $$\mathrm{Sin}\varphi =\frac{L}{\sqrt{L^{2}+l^{2}}}$$

(11) $$kbKT_{0}=0.67f_{cu}0.45\left(\frac{d_{1}+d_{2}}{2}\right)-\left(\frac{KT_{0}+T_{0}}{2}\right)$$

(12) $$b=\frac{1}{kKT_{0}}\left[0.67f_{cu}0.45\left(\frac{d_{1}+d_{2}}{2}\right)-T_{0}\left(\frac{K+1}{2}\right)\right]$$

where *KT*_0_ is the force per unit width in the steel along the short direction; *L* and *l* are the span and length of the rectangular plate, respectively; *k* is the parameter used to determine the magnitude of the membrane force (*k* = 1.4 based on the force equilibrium in Element 1); *φ* is the angle that defines the yield line pattern; *b* is the parameter used to determine the magnitude of the membrane force; and *d*_1_ and *d*_2_ are the effective depths of the short and long spans, respectively.

Einpaul [59] proposed the load–rotation relationship (Equation (13)) based on the critical crack theory, with a focus on the effect of moment redistribution on the CMA. However, this model provides insights into the self-constrained membrane action due to bending cracks at the slab–column connection, but it does not consider the constraint contribution from the supporting system, limiting its applicability to more complex structural conditions.

(13) $$\psi_{sc}=\left(1-\frac{2m_{cr}}{m_{R\cdot hog}}\right)\psi$$

where *ψ_sc_* is the rotation near the self-constrained plate connection; *m_cr_* is the cracking moment per unit plate width, and *m_cr_* = *f_ct_h*^2^/6; and *m_R·hog_* is the negative bending moment per unit plate width.

### 3.2. Punching Shear Mechanism

The punching shear mechanism of the slab–column joints is a fundamental aspect in the design of concrete structures. Its essence lies in coordination of the interaction between bending moments, shear forces, and material strengths. In 1907, Talbot [60] proposed the first punching shear calculation formula based on stress distribution through experiments on 200 wall–column connections, laying the foundation for subsequent research.

**Figure 12:**
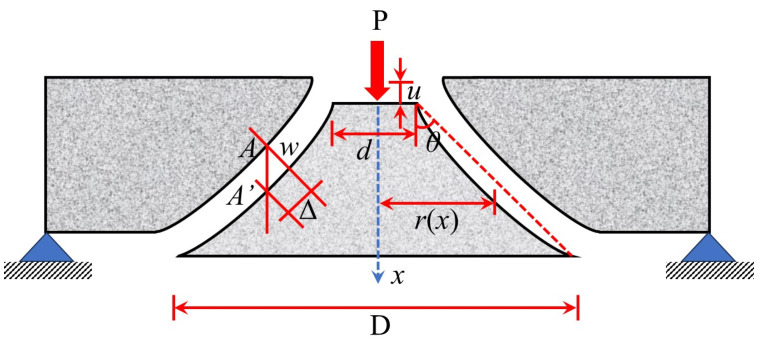
Plastic limit theory model.

**Figure 13:**
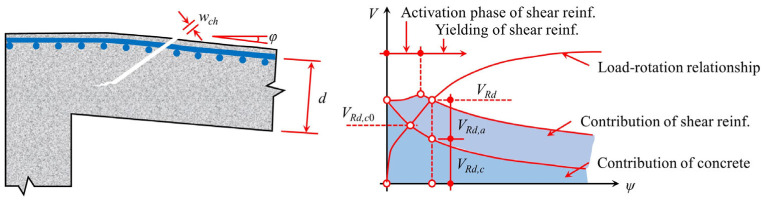
CSCT model.

The plastic limit theory proposed by Braestrup [61] utilizes the linear Mohr–Coulomb criterion [62] and assumes the punching shear region to be a slanted cone (Figure 12). Although this theory can explain the punching failure mechanism of circular plates, the calculation results exhibit considerable scatter. Jiang et al. [63] improved this theory by adopting a quadratic parabolic yield criterion, significantly enhancing its general applicability. Bortolotti [64] also optimized this theory and found that plastic strain leads to concrete softening, resulting in changes to the local internal friction angle. Based on experimental data, Muttoni [65] proposed the Critical Shear Crack Theory (CSCT), which determines the ultimate load by identifying the intersection point of the crack width and the load–rotation curve (Figure 13). This theory comprehensively considers parameters such as plate thickness, reinforcement ratio, and aggregate size. Its shear stress distribution in the triangular form, along with the assumption of critical crack propagation, reveals the sudden nature of brittle failure. However, while this theory provides a more comprehensive approach, it still assumes idealized behavior that may not fully reflect real-world conditions. The calculation method is shown in Equation (14).

(14) $$\frac{V_{R}}{b_{0}d\sqrt{f_{c}^{\prime }}}=\frac{3/4}{1+15\frac{\psi d}{d_{g0}\;+\;d_{g}}}$$

where *ψd* is the nominal crack width, and the calculation of *ψ* is given by Equation (15); *d* is the plate section height; *d*_g_ is the maximum aggregate size; and *d*_g0_ is the relative size of the aggregate particle, and *d*_g0_ = 16 mm.

(15) $$\psi =1.5\frac{r_{s}}{d}\frac{f_{y}}{E_{s}}{\left(\frac{V}{V_{\mathrm{flex}}}\right)}^{3/2}$$

where *r*_s_ is the radius of the plate; *f*_y_ is the yield strength of the longitudinal reinforcement; *E*_s_ is the elastic modulus of the reinforcement; *V*_flex_ is the bending capacity obtained from the yield line theory, and *V*_flex_ = 8*m*_Rd_; and *m*_Rd_ is the ultimate bending moment per unit plate width, and *m*_Rd_ = ${\rho d}_{v}^{2}$*f*_y_ (1 − *ρf*_y_/2*f*_c_).

Regarding the triaxial stress state, 68. Reineck [66] proposed a tensile stress model emphasizing the dominant role of the principal tensile stress in punching shear failure. While this model provides valuable insights, it assumes a simplified stress state and does not account for the complex interactions present in actual slab–column connections. Menetrey [67] optimized this model by refining the triaxial compression assumption at the column edge, enhancing its applicability. However, this modification still relies on idealized material behavior and may not fully capture the nonlinearities observed in experimental studies. The pressure path theory, extended by Kotsovos [68,69], introduced a dual plate strip stress transfer mechanism and identified horizontal cracking in the triaxial compression zone as a failure trigger. This approach offers a more comprehensive understanding of stress distribution but requires further validation through experimental data to confirm its accuracy and applicability in diverse structural configurations. The sectional strain model proposed by Park-Choi [70] focuses on the complex stress state in the concrete compression zone of the critical section, revealing the correlation between bending cracking and punching shear failure. These theories offer different perspectives on the stress evolution in the joint zone, but all require experimental data to validate their applicable boundaries.

### 3.3. Post-Punching Shear Mechanism

**Figure 14:**
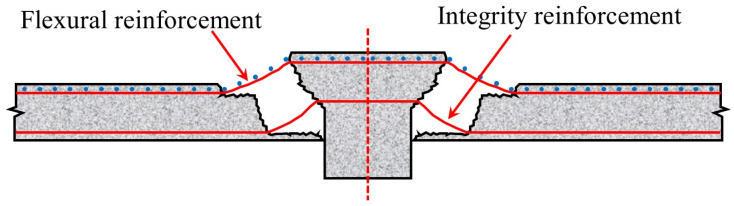
Schematic diagram of punching shear failure in flat slab–column joints.

After the punching shear failure in flat slab–column joints, the bending reinforcement and integrity reinforcement become the key force transfer paths linking the punching cone to the floor slab (Figure 14). These reinforcements are gradually activated under sustained loading, forming the “last line of defense against progressive collapse” and delaying the overall failure process through the membrane action mechanism [71].

**Figure 15:**
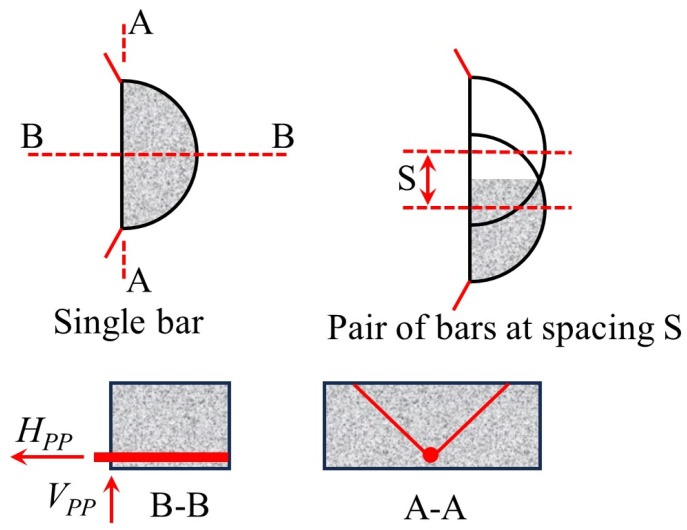
The analysis model proposed by Melo and Regan [49].

Regan [71], based on Rasmussen’s pinning action theory [72], proposed Equation (16), quantifying the post-punching shear strength contribution of integrity reinforcement through the reinforcement diameter, yield strength, and concrete compressive strength. While this model provides useful insights into the role of integrity reinforcement, it assumes idealized material behavior and does not account for complex interactions between reinforcement and concrete under actual loading conditions. Building on this, Georgopoulos [73] introduced the eccentricity parameter and established Equations (17) and (18) to predict the strength of the compressive reinforcement and the failure rotation. However, experiments by Melo and Regan [49] showed deviations in this model, indicating that it may not fully capture the real behavior of slab–column connections under punching shear (Figure 15). This prompted the adoption of ACI 349 code [74], which proposed Equations (19) and (20). In this approach, the resistance of the bottom reinforcement is considered as the pullout effect of concrete anchorage, and the calculated results closely match the experimental peak strength. Simultaneously, Equation (21) was introduced to account for the weakening effect of reinforcement fracture on the ultimate resistance, further improving the model’s accuracy. However, this modification still requires experimental validation to confirm its applicability in different structural configurations.

(16) $$V_{pp.\mathrm{int}.\;}=1.2\sum \Phi^{2}\sqrt{f_{sy}f_{c}}$$

where *V_pp_*._int_. is the contribution of the overall reinforcement to the post-punching shear resistance; Φ and *f_sy_* are the diameter and yield stress of the overall reinforcement, respectively; and *f* is the concrete compressive strength.

(17) $$V_{DU}=B\phi^{2}\sqrt{f_{c}f_{sy}}$$

(18) $$\sin\psi_{\mathrm{int}\;}=1.5\sqrt{\frac{f_{c}}{f_{sy}}}$$

where *e* is the eccentricity of the load; $B=C\left(\sqrt{1+{\left(\zeta C\right)}^{2}}-\zeta C\right)$; $\zeta =3e\sqrt{f_{c}/f_{sy}}/\phi$.

(19) $$V_{R.pp}=0.33\sqrt{f_{c}^{\prime }}\pi \frac{d_{res}^{2}}{2}$$

(20) $$V_{R.pp}=0.33\sqrt{f_{c}^{\prime }}\pi \frac{d_{res}^{2}}{2}-A_{1}$$

where $A_{1}=(\Delta \pi d^{2}/360)-(sd_{res}\sin\Delta /4)$; $\Delta =\arccos\left(s/2d_{res}\right)$.

(21) $$V_{R.pp.rupt}=0.44\sum A_{s}f_{y}$$

**Figure 16:**
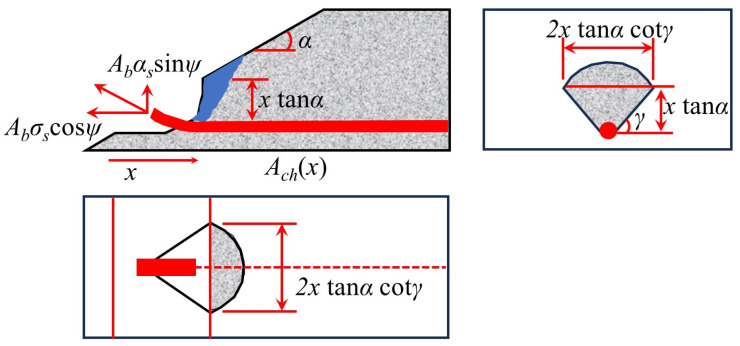
Concrete failure after punching shear [7].

Mirzaei [7] and Ruiz et al. [11] further considered the synergistic effect between bending and integrity reinforcement, and proposed Equations (22)–(27). The model assumes that the upper concrete of the reinforcement experiences continuous failure (Figure 16) and establishes the plastic hinge development equation (Equation (25)) and the strain–rotation relationship (Equations (26) and (27)). While this approach offers a more comprehensive view of the interaction between bending and integrity reinforcement, it relies on idealized failure assumptions, particularly the assumption of continuous failure in the upper concrete, which may not fully reflect actual conditions. Ruiz et al. [11] systematically quantified the coupled effects of bending reinforcement, integrity reinforcement, and concrete parameters using Equations (29)–(38), with Equations (30)–(35) focusing on the relationship between tensile reinforcement contribution and geometric parameters. While these equations provide valuable insights into the interaction of different factors, they may oversimplify complex behaviors that require further experimental validation. Habibi et al. [75] proposed Equation (28), which uses a dual inclination angle hypothesis of 45° and 14°, but only considers the reinforcement membrane mechanism, resulting in an insufficient evaluation of concrete failure severity. This limitation reduces the model’s accuracy in capturing the full extent of punching shear failure, highlighting the need for further refinement.

(22) $$V_{\mathrm{con}\;}(x)=\frac{\pi }{2}{\left(x\tan\alpha \cot\gamma \right)}^{2}f_{\mathrm{ct}.\mathrm{eff}\;}$$

where *α* is the inclination of the punching cone relative to the horizontal direction; *γ* is the angle of the punching cone; and *f*_ct_._eff_ is the effective tensile strength of concrete, and *f*_ct_._eff_ is given by *n_D_f_ct_*, where *n_D_* is a reduction factor that accounts for the variation of tensile stress from the edge of the reinforcement to the surface of the slab. If there are *n* reinforcement bars, the total area of the affected region is given by Equations (23) and (24).

(23) $$A_{1}=4\left\{\left[\Delta +\frac{n}{2}(\pi -2\Delta )\right]d_{\mathrm{res}\;}^{2}+\frac{n-1}{2}sd_{\mathrm{res}\;}\sin\Delta \right\}$$

(24) $$V_{s.\mathrm{Elas}.\;}=A_{b}E_{s}\sin\psi +V_{I}\cos\psi$$

where *A_b_* and *E_s_* are the cross-sectional area and elastic modulus of the reinforcement, respectively; *V_I_* is the shear at the reinforcement cross-section. For the development of plastic hinges near the concrete punching cone and the plate–node connection, the calculation is performed using Equation (25).

(25) $$V_{s.\mathrm{Plas}.\;}=N_{1}\sin\psi +\frac{\Phi^{3}}{3l_{re}}f_{sy}\left(1-\frac{N_{1}^{2}}{N_{y}^{2}}\right)\cos^{2}\psi$$

where *N*_1_ and *N_y_* are the axial forces in the reinforcement during the elastic and yielding stages, respectively; *l_re_* is the exposed length of the reinforcement. Additionally, the strain and rotation at failure must be considered.

(26) $$\varepsilon_{su}=\frac{1}{\cos\psi_{\max}}-1$$

(27) $$\tan\psi_{u}=\frac{w_{u}}{l_{re}}$$

(28) $$V_{s.\mathrm{reinf}.\;}=N_{1}\sin\psi$$

(29) $$V_{R,pp}=V_{R,pp,flex.}+V_{R,pp,int}$$

(30) $$V_{R,pp,flex.}=n_{b,flex}\cdot f_{ct}\cdot b_{ef}\cdot l_{ef}$$

(31) $$n_{b,flex.}=4\cdot n_{a}=4\cdot \frac{c+2d\cot\theta }{s_{b}}$$

(32) $$\cot\theta =\frac{\cot\theta_{top}+\cot\theta_{\mathrm{bottom}\;}}{2}$$

(33) $$f_{ct}≅0.5\sqrt{f_{c}}(MPa)$$

(34) $$b_{ef}=\min\left(s_{b}-d_{b};6d_{b};4c_{b}\right)$$

(35) $$l_{ef}=2d_{b}$$

(36) $$V_{R,pp,int}\leq f_{ct}\cdot A_{c,ef}$$

(37) $$A_{c,ef}=d_{res}\cdot b_{int}$$

(38) $$b_{\mathrm{int}\;}=\sum \left(s_{b,\;\mathrm{int}\;}+\frac{\pi }{2}d_{\mathrm{res}\;}\right)$$

where *V_R,pp_* is the post-punching shear strength at the flat slab–column joint; *V_R,pp,flex_.* is the contribution of the tensile reinforcement, as given by Equation (30); *V_R,pp,int_* is the contribution of the full reinforcement’s resistance, as provided by Equation (36). Equations (30)–(35) are used to determine the calculation formulas for the various parameters of *V_R,pp,flex_.*; *n_b,flex_.* is the number of bending reinforcement sections that participate in the action after the punching failure; *f_ct_* is the tensile strength of the concrete; *b_ef_* and *l_ef_* are the effective width and length of the concrete, respectively; *c* is the dimension of the column; *d* is the effective depth of the slab; *θ* is the average angle of the bending reinforcement’s action point; *c_b_* is the concrete cover thickness; *d_b_* is the diameter of the reinforcement; and *s_b_* is the spacing of the reinforcement.

## 4. Numerical Simulation of Progressive Collapse in Flat Slab–Column Structures

Due to the unique structural characteristics of the flat slab–column joints, researchers have successfully replicated the chain reaction process from local punching shear failure at the node to the overall collapse of the floor using numerical simulation methods. In this process, numerical simulations are capable of accurately capturing the concrete damage-softening behavior and the bonding effect between the reinforcement and the concrete. However, balancing computational efficiency with model accuracy, as well as verifying parameter sensitivity under various loading and interaction conditions, remains a core challenge that needs to be addressed in current numerical simulation research.

### 4.1. Types of Simulation Methods

Various simulation methods have been employed by researchers, such as the Finite Element Method (FEM), the Discrete Element Method (DEM), and both explicit and implicit integration methods, to analyze and optimize the collapse process of flat slab–column structures.

**Figure 17:**
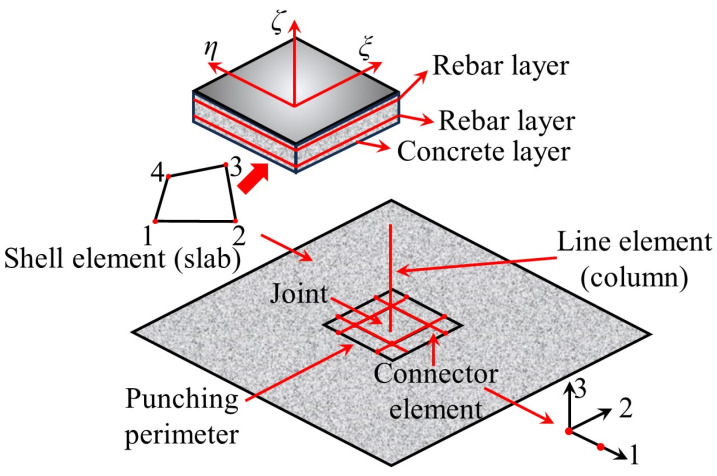
Macro-scale finite element model [76].

To reduce computational effort, Liu et al. [76,77] referred to simplified modeling methods such as the grid model [78,79,80], equivalent frame, and equivalent beam method [81,82,83,84,85,86,87,88] and proposed a “macro-scale finite element model” (Figure 17) that includes connection elements and shell elements to simulate the nonlinear response and node punching shear failure of flat slab–column structures under complex loading conditions. The model uses shell elements to simulate the bending performance and load redistribution characteristics of the slab and connection elements to simulate the separation that occurs at the flat slab–column joint during punching shear failure. Additionally, the model defines a deformation-based punching shear failure criterion for the connection elements to simulate the punching shear failure and load transfer at the flat slab–column joint. The simulation results indicate that using the shell elements can accurately predict the load–displacement response of the structure. However, due to the assumption of constant concrete shear stiffness, the exclusive use of shell elements tends to lead to an overestimation of the torsional resistance of the flat slab–column joint, resulting in an overestimation of the joint’s ability to transfer unbalanced bending moments.

### 4.2. Local Joint Model

**Figure 18:**
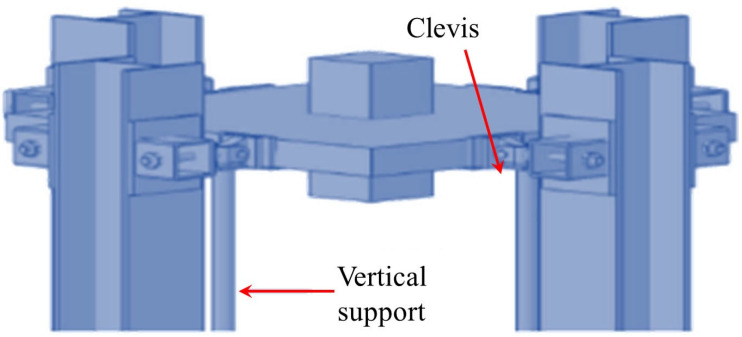
Flat slab–column joint model [89].

**Figure 19:**
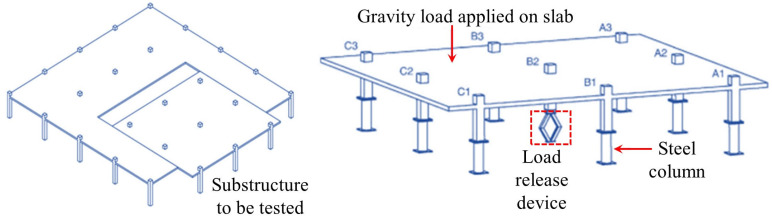
Punching shear model for the flat slab–column joints [90].

Peng et al. [89] simulated the load-carrying performance of unconstrained and constrained flat slab–column joints under concentrated loads (Figure 18) based on the macroscopic model proposed by Liu et al. [76,77]. This model uses shell elements and connection elements to simulate the nonlinear behavior of the slab, where the rigid shell elements are employed to model the flat slab–column joint region, and the slab surrounding the joint is modeled with shell elements that define the reinforcement layers. The separation between the slab and the column during punching shear failure is simulated using connection elements. The numerical simulation results indicate that compressive membrane action generates transverse constraints within the slab, which significantly enhance the structure’s resistance to progressive collapse. Mousapoor et al. [90] employed Liu et al.’s [76,77] macroscopic model within OpenSees to establish a punching shear model for flat slab–column joints (Figure 19). In this model, the slab is represented by quadrilateral planar shell elements (NLDKGQ), which, using the updated Lagrangian formulation, can effectively simulate the geometric nonlinearity in large structural deformations [91,92]. To focus on modeling damage failure at the flat slab–column connection, Mousapoor et al. [90] used “node-to-node connection” elements at the joint interface, where each node of the connection element has six degrees of freedom, including three translational and three rotational degrees of freedom. The results show that this modeling method provides good prediction accuracy.

### 4.3. Global Structural Model

**Figure 20:**
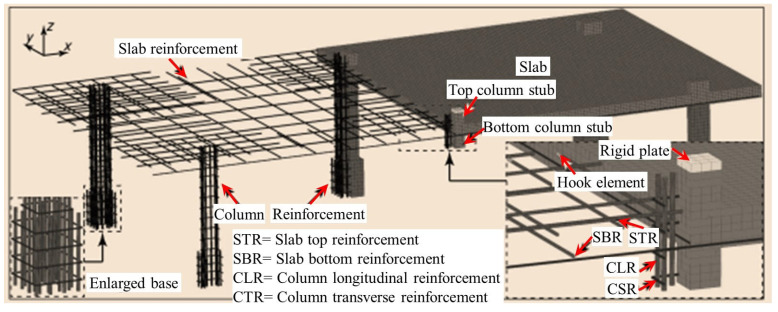
Bond–slip model for flat slab–column structures [93].

**Figure 21:**
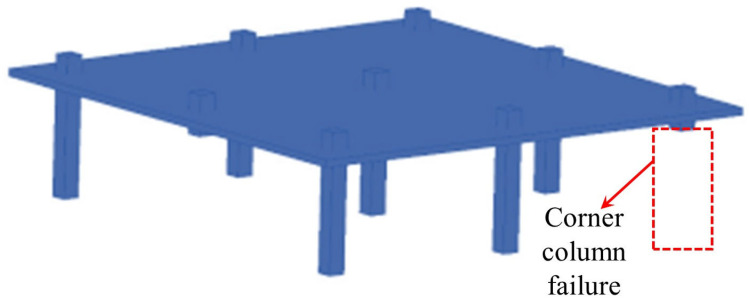
Separate model for reinforced concrete flat slab–column structures.

**Figure 22:**
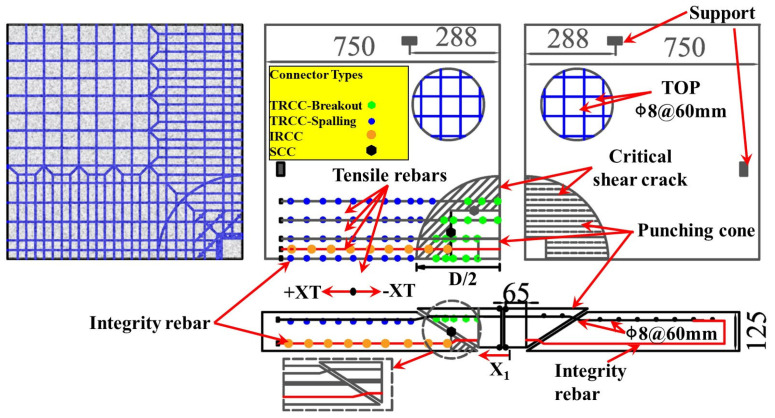
Continuous/separate model for punching shear failure in slab-shell elements before and after failure [42].

Overall, modeling techniques demonstrate the load-transfer behavior of flat slab–column structures in progressive collapse through multiscale methods. Xue et al. [93] established a bond–slip finite element model (Figure 20) using LS-DYNA to simulate secondary punching shear failure at the joint by reducing the bending stiffness and verified the significance of the bond effect on post-punching performance. Bu et al. [94] employed a solid-beam element separation model (Figure 21) to compare various column failure scenarios, finding that collapse resistance was optimal when the middle column failed, followed by edge columns, with corner columns being the weakest. Keyvani et al. [42] constructed continuous/separated models in ABAQUS (Figure 22), simulating flat slab–column joint separation using Cartesian–Cardan connection elements, and confirmed that axial constraints at the slab edges form a compressive membrane mechanism, enhancing punching shear strength by 34%. Additionally, the anchorage quality of the integral reinforcement plays a key role in the collapse resistance.

**Figure 23:**
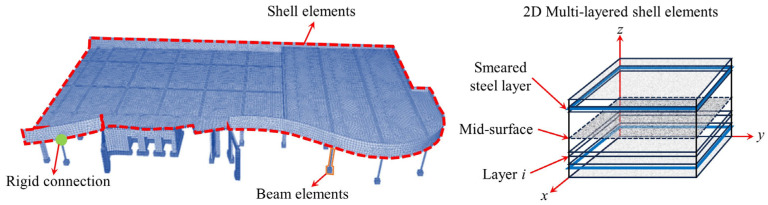
Flat slab–column structure model for parking garage roofs [95].

**Figure 24:**
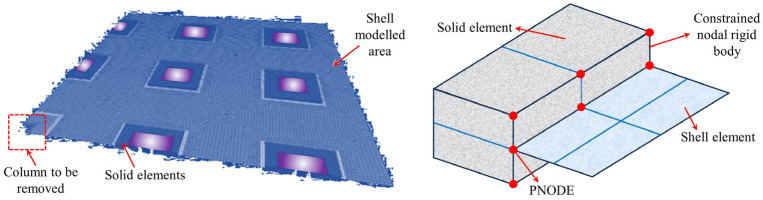
Dynamic numerical model for flat slab–column structures [97].

Halvoník et al. [95] employed SOFiSTiK to establish a garage roof model (Figure 23), incorporating the CSCT theory [96] to evaluate punching shear strength and accurately simulate both linear elastic and nonlinear responses. Ulaeto et al. [97] developed a solid-shell coordination constraint model (Figure 24) to ensure consistency between damage prediction and analytical methods. Their study showed that the void mechanism enhances stability by redistributing axial forces [18,98], and multilevel modeling effectively captures the constraint effects between upper and lower layers.

**Figure 25:**
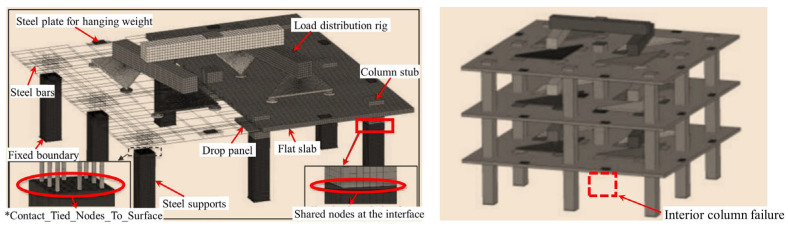
Refined model for column failure in multi-story flat slab–column structures [99].

**Figure 26:**
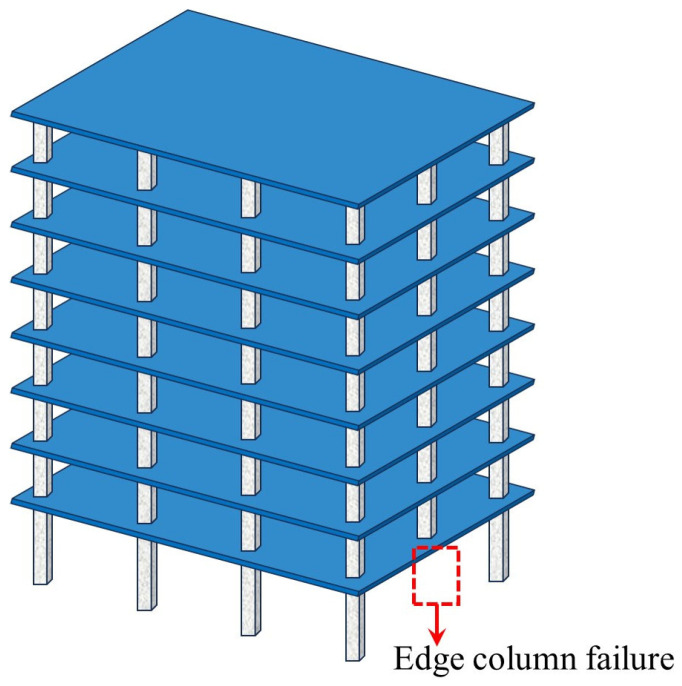
Stress contour map for multi-story unbonded prestressed flat slab–column structures.

**Figure 27:**
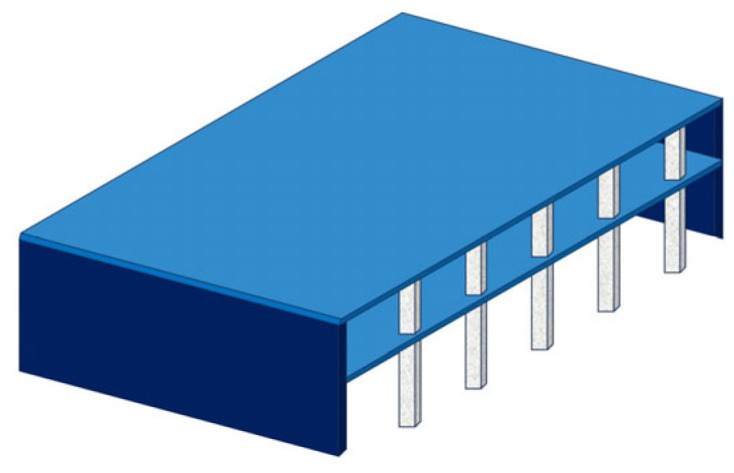
Two-story flat slab–column structure model for parking garage.

**Figure 28:**
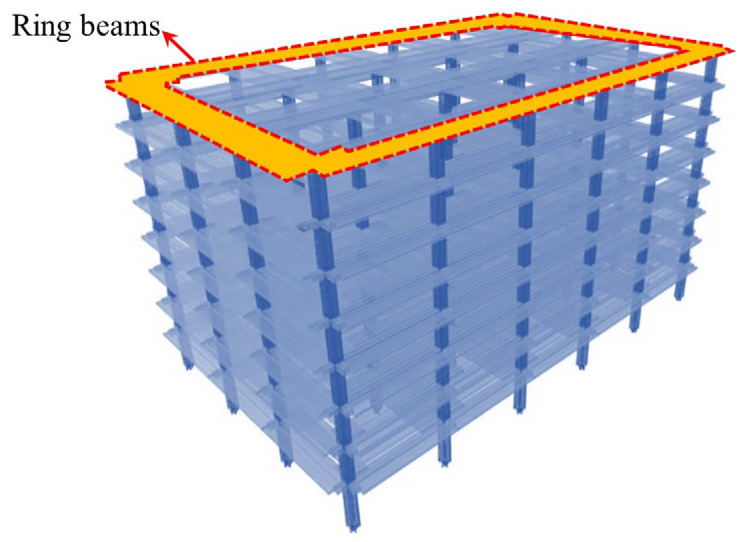
Integrity model for an 8-story reinforced concrete flat slab–column structure [102].

Weng et al. [99] developed a refined model for the middle column failure using LS-DYNA (Figure 25), employing the Continuous Surface Cap Model theory to predict punching shear failure and highlighting the differences in the resistance contributions from each floor. Cheng et al. [100] analyzed an eight-story prestressed structure using the ABAQUS CDP model (illustrated in Figure 26), incorporating CSA [14] and DoD [15] standards to verify the collapse mode under column removal scenarios. Cosgun et al. [101] developed a parking garage model using SAP2000 (Computers and Structures, Inc., Berkeley, CA, USA) (illustrated in Figure 27), demonstrating that insufficient shear reinforcement and design errors significantly decrease collapse resistance. Garg et al. [102] compared the effects of ring beams with ETABS (Figure 28), finding that the inclusion of ring beams enhances redundancy under various column removal scenarios.

## 5. Summary and Outlook

Research on the progressive collapse resistance of reinforced concrete flat slab–column structures has established a relatively complete theoretical framework for the punching shear failure mechanism at the joints. However, the key challenges remain, and future directions for studying collapse resistance at the overall structural level have emerged:

(1) Dynamic collapse mechanism and design methods: current research primarily relies on static experiments, which are inadequate in reflecting the impact of dynamic amplification effects (such as inertial forces and strain rate sensitivity) on structural performance during the collapse process. Dynamic loading experiments are required to reveal the load transfer paths and energy dissipation patterns, develop resistance assessment methods that consider dynamic responses, and enhance the calculation system for DAFs in engineering codes.
(2) Collaborative mechanism of multi-story structures: research findings from single-layer substructures cannot directly inform the design of multi-story systems. It is crucial to investigate the correlation mechanisms between interlayer shear redistribution, joint stiffness degradation, and longitudinal/transverse collapse modes. A multiscale analysis model, considering the void effect and redundancy evolution, must be developed to clarify the critical collapse transition conditions.
(3) Multi-hazard coupling effects: current research is limited to single-disaster scenarios. Systematic quantification of damage accumulation patterns under the coupling effects of multiple hazards, such as earthquakes, fires, and explosions, is necessary. Key challenges include material performance degradation due to high-temperature-shock coupling, post-disaster residual load-bearing capacity assessment, and the development of active protection technologies based on smart materials.
(4) Development of refined numerical models: current finite element models are inadequate in representing nonlinear behaviors such as large deformations and material fracture. There is a need to develop constitutive models that integrate physical information with neural networks to simulate concrete softening, reinforcement slip, and fracture efficiently. Additionally, a digital twin platform considering 3D-printed joint construction must be established to promote the intelligent integration of design, construction, and operation maintenance throughout the entire lifecycle.

## Data Availability

No data were used for the research described in the article.
